# Certain Associations Between Iron Biomarkers and Total and γ' Fibrinogen and Plasma Clot Properties Are Mediated by Fibrinogen Genotypes

**DOI:** 10.3389/fnut.2021.720048

**Published:** 2021-08-10

**Authors:** Petro H. Rautenbach, Cornelie Nienaber-Rousseau, Zelda de Lange-Loots, Marlien Pieters

**Affiliations:** ^1^Center of Excellence for Nutrition, North-West University, Potchefstroom, South Africa; ^2^Medical Research Council Unit for Hypertension and Cardiovascular Disease, North-West University, Potchefstroom, South Africa

**Keywords:** coagulation, ferritin, fiber density, fibrinolysis, lag time, lateral aggregation, transferrin, turbidity

## Abstract

**Introduction:** Evidence for the relationship between body iron and cardiovascular disease (CVD) is inconsistent and mechanisms involved remain poorly understood. Therefore, we first investigated whether there are linear or non-linear relationships between iron status and total and γ' fibrinogen as well as plasma fibrin clot properties and, second, determined whether there are interactions with iron biomarkers and fibrinogen and *FXIII* single nucleotide polymorphisms (SNPs) in relation to fibrinogen concentration and functionality.

**Methods:** In this cross-sectional analysis of 2,010 apparently healthy Black South Africans we quantified total and γ' fibrinogen, serum iron, ferritin and transferrin using standardized methods and calculated transferrin saturation (TS). Clot architecture and lysis were explored with a global analytical turbidity assay. The SNPs were determined through an Illumina BeadXpress^®^ platform.

**Results:** Total, but not %γ', fibrinogen negatively correlated with serum iron concentrations, although both decreased over iron tertiles. %γ' fibrinogen correlated negatively with transferrin and decreased over the transferrin tertiles. A weak negative association between total fibrinogen and TS was detected with fibrinogen decreasing over the TS tertiles and categories based on TS. Lag time correlated positively with transferrin and increased over transferrin tertiles, when adjusting for fibrinogen. Before adjusting for fibrinogen, lag time was shorter in those with adequate iron status based on TS than other iron subcategories. Clot lysis time (CLT) negatively correlated with ferritin and was longer in the first than in the third ferritin tertile. Among iron status categories based on ferritin, only CLT differed and was longer in those with adequate iron than with iron-overload. CLT negatively correlated with TS, albeit weakly, shortened over the TS tertiles and was shorter in those with adequate iron based on TS categories. Interactions were observed between *FGB* SNPs and some of the markers of iron status investigated, in relation to the clot properties with the most prominent associations detected in homozygous carriers of the variant alleles for whom increased iron status was more beneficial than for those harboring the wild-type alleles. Iron modulated the influence of the SNPs so that for the majority iron was beneficial in respect of clot properties, but even more so for a minority group harboring specific variant alleles.

**Conclusion:** This is the first large-scale epidemiological study to relate fibrinogen concentration and functionality to markers of iron status and to take genetic factors into consideration. We have detected a relationship between iron biomarkers and fibrinogen as well as clot characteristics that are influenced by the genetic make-up of an individual.

## Introduction

Cardiovascular disease (CVD) continues to soar on a trajectory that will cripple many health services (1), despite being preventable by appropriate lifestyle modification (2), including diet (3). Micronutrients (4) such as the dietary trace mineral, iron, may play a role in CVD (5, 6). In those with optimal iron status, iron has been shown to have a neutral effect, but CVD risk is modestly increased in those with a deficiency or in individuals with iron-overload (5, 7). The mechanisms for this association remain to be determined. However, plausible pathways include effects on fibrinogen concentration, blood clot structure and properties (8, 9), which have themselves been implicated in CVD (10–12). The circulating glycoprotein fibrinogen is, upon activation, converted to fibrin, which provides the matrices for blood clots. Furthermore, fibrinogen has a common splice variant, γ' fibrinogen, with a physiological range of 8–15% (13), known to influence fibrin clot structure and mechanics (14). Factor XIII (FXIII) is another constituent of blood, which crosslinks fibrin fibers to protect the clot against premature lysis to allow wound healing (15). Single nucleotide polymorphisms (SNPs) within the fibrinogen α, β and γ chain genes (*FGA, FGB*, and *FGG*) influence total and γ' fibrinogen concentrations (16, 17) and have also been demonstrated together with those of *FXIII* to influence clot properties (16, 17). Undesirable iron stores (due to either iron overload or iron deficiency) are associated with oxidative stress (18–21), which can lead to post-translational modifications, but have also been shown to direct binding of iron to the fibrinogen molecule, leading to conformational changes and thereby hypercoagulability (22–24).

There are various iron biomarkers, representing different components of iron status. The gold standard for measuring iron stores is to evaluate iron deposits in a bone marrow aspiration sample (25). However, this procedure, being invasive and expensive, is not feasible in epidemiology (5). Ferritin was asserted to be the most powerful indicator for simple iron deficiency in populations (26), being a storage protein which is proportional to total-body iron stores and decreases when the body needs more of this essential trace mineral. Transferrin is a transport protein, which increases when the body needs more iron to increase absorption. Concentrations of iron in serum represent the circulating iron bound to transferrin and ferritin. From the latter markers, transferrin saturation (TS) can be calculated to increase the accuracy of iron deficiency detection (27). TS signifies the total iron availability in the body (indicating deficiency, adequacy or overload) and the equilibrium between the release of iron from storage areas and its use by bone marrow for erythropoiesis (28). TS is deemed a good indicator of iron reserves in the bone marrow (29). We included both serum ferritin and TS in our investigation to provide the best possible assessment of iron status (27). Hemoglobin and hematocrit are late indicators of iron status (30) and dietary intake is inadequate as a sole indicator of depletion (31), therefore these criteria should be interpreted with caution.

The aim of this study was 2-fold: to increase our understanding of the manner in which iron stores associate with fibrinogen concentration and functionality (fibrin clot properties), and how these associations are influenced by specific variations within the fibrinogen and *FXIII* genes. To this end, we first determined linear and non-linear relationships between total fibrinogen and—to our knowledge for the first time—the γ' splice variant of fibrinogen, and clot properties and iron markers, in a large population-based study. Second, because fibrinogen and *FXIII* SNPs might interact with iron status markers to modulate total or γ' fibrinogen concentrations as well as the kinetics of clot formation, structural clot properties and fibrinolysis, we examined whether there were such interactions. This work could potentially contribute to personalized nutrition in which dietary recommendations are tailored to genetic makeup, to enhance the therapeutic success of diet-based lifestyle interventions on disorders contingent on hemostasis.

## Materials and Methods

### Study Design, Population Selection, and Demographic Characteristics

This cross-sectional research is nested within the South African arm of the *P*rospective *U*rban and *R*ural *E*pidemiology (PURE) study that forms part of a multinational investigation tracking risk factors for chronic non-communicable diseases (32). A three-stage sampling process was followed with sample size based on power calculations using a previous investigation (33). After obtaining gatekeeper permission from local authorities, 6,000 houses were randomly selected from urban and rural strata. From the households, 4,000 eligible individuals with no reported use of chronic medication (excluding that for blood pressure) or any self-reported acute illness were identified. During the baseline period (data reported in this paper), biological samples were collected from 1,006 rural and 1,004 urban, ostensibly healthy Black South African men and women, who provided written informed consent. The study protocol was approved by the Health Research Ethics Committee of the authors' University (NWU-00016-10-A1 and NWU-00034-17-A1-02), honoring the guidelines laid down in the Declaration of Helsinki of 1975 and revised in 2000.

### Lifestyle Factor Assessment

Questionnaires were interviewer-based and conducted by trained fieldworkers in the participant's home language. Details regarding demographic and lifestyle factors, including tobacco and alcohol use, were obtained. These results were published previously (34).

### Anthropometrical and Blood Pressure Assessment

Participants were weighed and their heights measured to calculate body mass index (BMI, kg/m^2^). Waist circumference was obtained at the narrowest point between the 10th rib and iliac crest via a measuring tape by level 1 anthropometrists. Blood pressure—systolic and diastolic—was measured with the OMRON HEM-757 apparatus (Omron Healthcare, Kyoto, Japan) with the cuff on the participant's left arm.

### Blood Collection, Sampling, and Storage

Fasting blood samples were collected by registered nurses between 07:00 and 11:00 from participants' antebrachial vein branches and centrifuged at 2,000 × g for 15 min. Sodium citrate-treated tubes were used to collect samples for the determination of fibrinogen concentration and clot properties. For high-sensitivity C-reactive protein (CRP), gamma glutamyl transferase (GGT) and lipids, tubes not containing anti-coagulants were used for blood collection. Ethylenediaminetetraacetic acid (EDTA) tubes were used for the quantification of serum ferritin, transferrin, iron and glycated hemoglobin A1c (HbA1c). Following removal of serum and plasma from the blood collection tubes, the remaining buffy coat was removed for the purpose of DNA isolation. Samples were transferred to collection tubes (500 μL), snap-frozen on dry ice and stored at −20°C for 2–4 days prior to transfer to the cryofreeze facilities at the NWU, where they remained at −80°C until analyzed.

### Biochemical Analyses

Total fibrinogen concentrations were quantified using the modified Clauss method on the Dade Behring BCS coagulation analyzer (Multifibrin U-test Dade Behring, Deerfield, IL, USA). An enzyme-linked immunosorbent assay (ELISA) using a Santa Cruz Biotechnology (Santa Cruz, Dallas, TX, USA) 2.G2.H9 mouse monoclonal coating antibody against human γ', and an Abcam (Cambridge, MA, USA) goat polyclonal horseradish peroxidase (HRP) conjugated antibody against human fibrinogen, were used to determine fibrinogen γ' concentrations. Fibrinogen γ' is expressed as % of total fibrinogen for the purpose of this paper.

To obtain plasma fibrinolytic potential, turbidimetric analysis (A405 nm) (35) with modified tissue factor and tissue-plasminogen activator (tPA) concentrations was used to obtain clot lysis times (CLT) of between 60 and 100 min. Final concentrations were: tissue factor (125x diluted—-an estimated final concentration of 59 pM; Dade Innovin, Siemens Healthcare Diagnostics Inc., Marburg, Germany), CaCl_2_ (17 mmol/L), tPA (100 ng/mL; Actilyse, Boehringer Ingelheim, Ingelheim, Germany) and phospholipid vesicles (10 μmol/L; Rossix, Mölndal, Sweden). Kinetics of clot formation (lag time and slope) and structural plasma fibrin clot properties (maximum absorbance) and CLT were calculated from the turbidity curves as described previously (36). Lag time represents the time required for fibrin fibers to attain a sufficient length to allow lateral aggregation and activation of the coagulation cascade. Slope reflects the rate of lateral aggregation and maximum absorbance indicates clot density.

Serum ferritin and transferrin concentrations were determined using an enzyme immunoassay procedure (Ramco Laboratories, Inc, Stafford, TX, USA) and serum iron, CRP and GGT were measured with a particle-enhanced immunoturbidimetric assay using a Sequential Multiple Analyzer Computer (SMAC) using the Konelab20iTM auto analyzer (Thermo Fisher Scientific Oy, Vantaa, Finland). Low-density lipoprotein cholesterol (LDL-C) was calculated using the Friedewald formula. HbA1c concentrations were quantified using the D-10 Hemoglobin testing system (Bio-Rad Laboratories, Hercules, CA, USA). Whole blood was used for the determination of HIV status, making use of the First Response rapid HIV card test (PMC Medical, India). If positive, the investigation was repeated with the Pareeshak card test (BHAT Bio-tech, India).

### DNA Extraction, Sequencing, and Genotyping

Genomic DNA was extracted using FlexiGene™ kits (QIAGEN, catalog number 51206). If insufficient yield was established, the Maxwell^®^ 16 blood DNA purification kit was used for the second isolation attempt. *FGA* 2224G/A (rs2070011), *FGA* 6534A/G (rs6050), *FGB* 1038G/A (rs1800791), *FGB* Arg448Lys, G/A (rs4220), *FGB* −148C/T (rs1800787), *FGG* 10034C/T (rs2066865), and *FGG* 9340T/C (rs1049636), the *FXIII* His95Arg A/G (rs6003) and *FXIII* Val34Leu, C/A (rs5985) were genotyped. Details of the genotyping are described elsewhere (16).

Quality control included a check for individuals successfully genotyped (missingness), minor allele frequencies (MAF), conformance to Hardy–Weinberg equilibrium (HWE) and a tally of Mendelian errors. Haploview version 4.2 software (37) was used to calculate the level of pairwise linkage-disequilibrium (LD) between the SNPs, as reported before (38). For this study population, three SNPs (rs7439150, rs1800787, and rs1800789) were in high, albeit not complete, LD. Therefore, we report the SNPs separately.

### Statistical Analysis

The computer software package *Statistica*^®^ version 13.3 (TIBCO Software Inc., Tulsa, OK, USA) and R version 3.5.0 (39) were used for computation. TS was calculated as follows (Fe/transferrin × 70.9 (40). A sensitivity analysis was included in which all statistical analyses were conducted before and after those with a CRP concentration ≥15 mg/L were excluded (this cut-off was used because the tympanic temperature of all participants included was <37.0°C, to rule out acute infection and the population is known to have a high basal CRP due to genetics (41) and socio-economic factors (42). However, because exclusion did not result in major differences, we opted to report the results for the whole population with adjustment for, rather than excluding, CRP.

The variables data were tested for normality using the Shapiro–Wilk W-test and the Kolmogorov–Smirnov test and descriptive statistics were calculated. Because variables were non-normally distributed, data are reported as medians (lower and upper quartiles).

Spearman correlations were performed to test for statistical dependence between variables. LDL-C, age, HbA1c, GGT and BMI were identified as possible confounders. We adjusted for these as well as CRP in subsequent analyses including in the Spearman partial correlations.

Kruskal–Wallis ANOVA and Mann–Whitney *U*-tests were used to detect differences in hemostatic markers among the tobacco use and HIV status subgroups as well as between men and women. Differences in total and %γ' fibrinogen and fibrin plasma clot properties (lag time, slope, maximum absorbance and CLT), among tertiles created for the iron biomarkers, ferritin categories (iron deficient <15 μg/L; adequate =15–≤200 μg/L; overload >200 μg/L) and TS categories (<20% iron deficient; ≥20% adequate) were analyzed using generalized linear models with adjustments and *post hoc* tests.

To investigate whether the biomarkers of iron status had interactive effects with the fibrinogen and *FXIII* SNPs in predicting total and γ' fibrinogen concentrations and clot properties, general linear models with adjustments and interactions were performed using the biomarkers as continuous variables. The low prevalence of homozygous carriers of the variant allele among the study participants did not allow further subdivision of the population into tertiles based on iron biomarkers within the interaction analyses. Interactions that remained after adjusting for multiple testing are reported in **Table 4**. The method of Benjamini-Hochberg (43) was used to account for multiple testing and false discovery rates in terms of the interactions. This procedure indicated that the statistical threshold for significance was *p* < 0.0017 [1/16*3*3(0.25)] when the false discovery rate was set at 25%. The number of SNPs investigated was 16, the iron biomarkers—based on associations with one another—were regarded as three, whereas total and γ' fibrinogen concentrations and fibrin clot properties were also regarded as three. To describe the interactions, rho values were determined through Spearman partial correlations adjusting for confounders including CRP and additionally for fibrinogen when the interaction was related to one of the clot properties.

## Results

The demographic and clinical characteristics of participants are presented in Table 1. We observed ferritin concentrations of <15 μg/L, indicative of iron deficiency (44) in 11.7% of the study population, and >200 μg/L, indicative of a risk of iron overload (44) in 26.7% of the volunteers; 39.4% presented with a TS of <20%, also indicative of iron deficiency (25, 44, 45).

**Table 1:** Specific characteristics of participants.

**Median (interquartile range) or (n: n)**			
			**Whole group^#^**
Sex (men: women)^*^			747: 1,263
**Age (yr)**			48.0 (41.0–56.0)
**Blood pressure (mmHg)**			
Systolic			130 (116–147)
Diastolic			87.0 (78.0–97.0)
**Tobacco use (%)^@^**			
Never (1)			52.7
Former (2)			4.14
Current (3)			42.7
**BMI (kg/m** ^**2**^ **)**			22.9 (19.3–28.6)
**Waist circumference (cm)**			77.5 (70.2–87.7)
**HIV status** 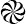			
(Unaffected: affected)			1,668: 326
**Biochemical markers**	**Cardiovascular and clot properties**		
		TC (mmol/L)	4.82 (4.01–5.87)
		LDL-C (mmol/L)	2.79 (2.08–3.65)
		HDL-C (mmol/L)	1.42 (1.06–1.87)
		Triglycerides (mmol/L)	1.08 (0.82–1.55)
		HbA1c (%)	5.50 (5.30–5.80)
		CRP (mg/L)	3.29 (0.96–9.34)
		Total fibrinogen (g/l)	2.90 (2.30–5.00)
		Fibrinogen γ' (%)	10.2 (7.14–14.6)
		Lag time (min)	6.52 (5.10–7.78)
		Slope (x10^−3^ AU/s)	8.90 (6.49–11.9)
		Maximum absorbance (nm)	0.42 (0.33–0.52)
		CLT (min)	57.2 (50.9–63.8)
	**Iron status biomarkers**		
	Iron (μmol/L)	Whole	15.9 (10.8–23.9)
		Tertile 1	9.30 (6.80–10.8)
		Tertile 2	15.9 (14.3–17.7)
		Tertile 3	28.5 (23.9–38.8)
	Transferrin (g/L)	Whole	2.72 (2.33–3.27)
		Tertile 1	2.18 (1.94–2.33)
		Tertile 2	2.71 (2.58–2.83)
		Tertile 3	3.56 (3.23–4.02)
	Ferritin (μg/L)	Whole	93.9 (36.4–208)
		Tertile 1	23.0 (11.5–36.4)
		Tertile 2	93.9 (72.7–121)
		Tertile 3	268 (208–445)
	TS (%)	Whole	23.08 (15.6–34.9)
		Tertile 1	13.0 (9.22–15.6)
		Tertile 2	23.1 (20.6–26.2)
		Tertile 3	34.9 (34.9–57.4)

# *Total sample was 2,010, but numbers are slightly different for several variables due to random missing data*.

* *Total fibrinogen, maximum absorbance and CLT differed between the sexes*.

@ *CLT differed among the tobacco use subgroups*.

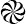
*Total fibrinogen, %γ' fibrinogen, slope and maximum absorbance differed between the HIV status groups*.

*BMI, body mass index; CLT, clot lysis time; CRP, high-sensitivity C-reactive protein; HbA1c, glycosylated hemoglobin A1c; HDL-C, high-density lipoprotein cholesterol; HIV, human immunodeficiency virus; LDL-C, low-density lipoprotein cholesterol; n, number of patients; TC, total cholesterol; TS, transferrin saturation*.

Spearman and partial Spearman correlations are presented in Table 2. Noteworthy correlations observed were a negative association of serum iron with total fibrinogen, and a positive association between TS and total fibrinogen. TS also correlated negatively with CLT. Most of the correlations observed between clot properties and markers of iron status disappeared after adjustment for fibrinogen. In Table 3 we divided the iron biomarkers into tertiles to explore their relationship with fibrinogen and clot structure.

**Table 2:** Correlations (r) of biomarkers of iron status with total fibrinogen and γ' fibrinogen concentrations and clot properties.

**Participant characteristics**	**Total fibrinogen**	**%γ'fibrinogen**	**Lag time**	**Slope**	**Maximum absorbance**	**CLT**
Iron (μmol/L)	−0.22^***^−0.12^**^ –	−0.04 −0.03 –	0.03 −0.02 −0.009	−0.13^***^ −0.08^*^ −0.05	−0.08^**^ 0.05 0.08*	−0.01^***^ 0.02 0.02
Transferrin (g/L)	0.06^**^ 0.03 –	−0.09^***^ −0.06 –	0.016 0.09^*^ 0.09*	0.03 −0.02 −0.02	0.08^***^ 0.06 0.05	0.12^***^ 0.01 0.01
Ferritin (μg/L)	0.06 0.09^*^ –	−0.05 −0.07 –	−0.02 −0.05 −0.06	0.008 0.04 0.02	−0.03 −0.03 −0.06	−0.1^**^ −0.1^**^ −0.1**
TS (%)	0.24^***^−0.1^**^ –	−0.002 0.02 –	0.03 −0.04 −0.02	−0.13^***^ −0.06 −0.03	−0.11^***^ 0.03 0.06	−0.15^***^ 0.01 0.02

* *p < 0.05;*

** *p ≤ 0.01;*

*** *p ≤ 0.001*.

*The first r-value is for Spearman correlation; second r-value for Spearman partial correlations of iron biomarkers with clot properties, adjusting for CRP, LDL-C, age, HbA1c and GGT; third r-value is for Spearman correlations of iron biomarkers with clot properties, adjusting for CRP, LDL-C, age, HbA1c and GGT as well as fibrinogen*.

*CLT, clot lysis time; TS, transferrin saturation*.

**Table 3:** Iron biomarker tertiles in relationship to total and γ' fibrinogen as well as clot properties (i.e., lag time, slope, maximum absorbance, and CLT).

	**Adjusted means with 95% CI determined through GLM**			
**Serum iron Model 1**	**<12.4 (μmol/L)**	**12.4–20.45 (μmol/L)**	**>20.45 (μmol/L)**	***p*** **-value**
Fibrinogen (g/L)	3.94 (3.79–4.10)*^#$^	3.70 (3.54–3.85)*^#$^	3.41 (3.25–3.57)*^#$^	0.00004
Fibrinogen γ' (%)	13.1 (12.4–3.83)*^#^	11.7 (11.1–2.43)*	11.5 (10.8–2.23)^#^	0.004
Lag time (min)	6.52 (6.36–6.68)	6.55 (6.40–6.70)	6.36 (6.21–6.52)	0.23
Slope (x 10^−3^AU/s)	9.74 (9.39–10.1)	9.56 (9.22–9.90)	9.64 (9.29–10.0)	0.76
Maximum absorbance (nm)	0.44 (0.42–0.45)	0.43 (0.42–0.45)	0.42 (0.41–0.44)	0.35
CLT (min)	58.6 (57.7–9.52)	57.0 (56.1–57.9)	56.1 (55.2 −57.0)	0.001
**Model 2**				
Lag time (min)	6.45 (6.29–6.61)	6.46 (6.30–6.62)	6.36 (6.20–6.52)	0.66
Slope (x 10^−3^ AU/s)	9.57 (9.22–9.91)	9.50 (9.17–9.84)	9.81 (9.46–10.15)	0.45
Maximum absorbance (nm)	0.43 (0.41–0.44)	0.43 (0.41–0.44)	0.43 (0.42–0.44)	0.97
CLT (min)	58.6 (57.6–59.5)	57.3 (56.4–58.2)	56.3 (55.3–57.2)	0.004
**Transferrin Model 1**	**<2.46 (g/L)**	**2.46–3.04 (g/L)**	**>3.04 (g/L)**	
Fibrinogen (g/L)	3.58 (3.39– 3.71)	3.67 (3.51–3.84)	3.74 (3.59–3.90)	0.20
Fibrinogen γ' (%)	13.3 (12.6–14.0)*^#^	11.8 (11.1–12.6)*	11.4 (10.8–12.1)^#^	0.0006
Lag time (min)	6.43 (6.30–6.60)	6.41 (6.25–6.58)	6.60 (6.44–6.75)	0.23
Slope (x 10^−03^ au/s)	9.35 (9.00–9.70)	9.96 (9.60–10.3)	9.51 (9.17–9.85)	0.05
Maximum absorbance (nm)	0.42 (0.41–0.43)	0.43 (0.42–0.45)	0.43 (0.42–0.44)	0.35
CLT (min)	57.2 (56.2–8.09)	57.1 (56.1–8.02)	57.5 (56.6–8.35)	0.83
**Model 2**				
Lag time (min)	6.35 (6.19–6.52)*	6.31 (6.14–6.48)^#^	6.58 (6.43–6.74)*^#^	0.04
Slope (x 10^−3^ AU/s)	9.36 (9.01–9.72)	9.92 (9.57–10.3)	9.47 (9.15–9.80)	0.07
Maximum absorbance (nm)	0.42 (0.40–0.43)	0.42 (0.41–0.44)	0.43 (0.42–0.44)	0.43
CLT (min)	57.4 (56.4–58.4)	57.2 (56.3–58.2)	57.5 (56.6–58.4)	0.92
**Ferritin Model 1**	**<54.9 (μg/L)**	**54.9–152.6 (μg/L)**	**>152.6 (μg/L)**	
Fibrinogen (g/L)	3.95 (3.73–4.16	3.91 (3.70–4.13)	3.78 (3.56–4.00)	0.54
Fibrinogen γ' (%)	13.1 (12.1–4.11)	12.1 (11.1–3.12)	12.5 (11.5–13.5)	0.37
Lag time (min)	6.53 (6.33–6.73)	6,20 (6.00–6.40)	6.44 (6.24–6.64)	0.03
Slope (x 10^−3^ AU/s)	9.71 (9.25–10.2)	9.71 (9.26–10.2)	9.82 (9.36–10.3)	0.93
Maximum absorbance (nm)	0.46 (0.44–0.47)	0.44 (0.43–0.46)	0.42 (0.40–0.44)	0.06
CLT (min)	60.1 (58.9–61.2)*	58.6 (57.4–59.7)	57.0 (55.9–58.1)*	0.002
**Model 2**				
Lag time (min)	6.54 (6.33–6.75)	6.16 (5.95–6.36)	6.32 (6.11–6.53)	0.04
Slope (x 10^−03^ au/s)	9.68 (9.21–10.1)	9.64 (9.19–10.1)	9.84 (10.1–9.38)	0.81
Maximum absorbance (nm)	0.45 (0.43–0.46)	0.44 (0.43–0.46)	0.42 (0.40–0.44)	0.09
CLT (min)	60.2 (59.0–61.4)*	58.4 (57.3–59.6)	57.4 (56.2–58.6)*	0.005
**TS Model 1**	**<18.02 (%)**	**18.02–30.01 (%)**	**>30.01 (%)**	
Fibrinogen (g/L)	3.97 (3.82–4.13)*^#$^	3.64 (3.48–3.91)*^#$^	3.42 (3.26–3.58)*^#$^	0.00001
Fibrinogen γ' (%)	3.97 (3.82–4.13)	3.64 (3.48–3.79)	3.42 (3.26–3.58)	0.4
Lag time (min)	12.7 (12.0–13.4)	11.6 (10.9–12.3)	12.1 (11.4–12.8)	0.54
Slope (x 10^−3^ AU/s)	9.78 (9.44–10.1)	9.57 (9.23–9.91)	9.58 (9.23–9.93)	0.64
Maximum absorbance (nm)	0.43 (0.42–0.45)	0.44 (0.42–0.45)	0.42 (0.41–0.43)	0.07
CLT (min)	58.7 (57.8–59.6)*^#$^	57.2 (56.3–58.0)*^#$^	55.8 (54.9–56.7)*^#$^	0.0009
**Model 2**				
Lag time (min)	6.41 (6.25–6.57)	6.46 (6.30–6.62)	6.40 (6.24–6.56)	0.86
Slope (x10^−3^ AU/s)	9.59 (9.26–9.93)	9.57 (9.24–9.90)	9.69 (9.34–10.0)	0.9
Maximum absorbance (nm)	0.43 (0.42–0.44)	0.43 (0.42–0.44)	0.42 (0.41–0.43)	0.41
CLT (min)	58.6 (57.6–59.5)*^#$^	57.4 (56.5–58.3)*^#$^	56.1 (55.2–57.1)*^#$^	0.002

*The first model for each iron biomarker is adjusted for age, LDL-C, BMI, HbA1c, GGT, and CRP, and the second additionally for fibrinogen. All tertiles contain ~670 individuals. Bonferroni post hoc test revealed significant differences between the categories indicated with symbols. CI, confidence interval; CLT, clot lysis time; GLM, general linear models; TS, transferrin saturation*.

Total fibrinogen but not γ' fibrinogen negatively correlated with serum iron concentrations, although both decreased over iron tertiles. %γ' fibrinogen correlated negatively with transferrin and decreased over the transferrin tertiles. A weak negative association between fibrinogen and TS was detected, with total fibrinogen decreasing over the TS tertiles. Lag time correlated positively, albeit weakly, with transferrin and increased over transferrin tertiles after adjustment for fibrinogen. Despite lag time not being correlated with ferritin, we detected a difference therein among ferritin tertiles. The negative correlation between circulating iron and CLT was weak and the decrease in CLT over the serum iron tertiles disappeared after *post hoc* tests. CLT negatively correlated with ferritin and was longer in the first than in the third ferritin tertile. CLT negatively correlated with TS, albeit weakly, and shortened over the TS tertiles. However, because clinical guidelines regarding iron status based on ferritin and TS cut-offs exist, we also report the significant differences observed among and between these categories. Among iron status categories based on ferritin, fibrinogen concentrations tended to be higher in the iron-adequate and overload groups than in the deficient group (*p* = 0.057) and CLT was shorter in the iron overload group than in those with an adequate status (*p* = 0.017). Between iron status categories based on TS, total fibrinogen (*p* = 0.00003), maximum absorbance (*p* = 0.02; after additional adjustment for fibrinogen *p* = 0.09) and CLT (*p* = 0.00008; after additional adjustment for fibrinogen *p* = 0.002) were lower in the iron-deficient than the iron-adequate group.

In Table 4 only the significant interactions (*p* < 0.0017) between the SNPs and the biochemical markers of iron status are presented in relation to fibrinogen as well as plasma clot structure and function. Serum iron interacted with the *FGB* SNP at rs4463047 in relation to slope. Participants harboring the wild type at this locus displayed an inverse, albeit weak, correlation between the rate of lateral aggregation and concentrations of serum iron, whereas slope and iron did not correlate in carriers of the variant allele. Ferritin interacted with the *FGB* SNP at rs2227388 in relation to lag time. Among the different genotypes at this locus, only heterozygous individuals presented with a negative relationship between ferritin and lag time, with the homozygous individuals of the minor allele being under-represented. A negative association, albeit weak, was seen with carriers of the wild-type genotype of the *FGB* SNP at rs4463047 between TS and slope, whereas there was none in individuals with the minor allele. The *FGB* SNP at rs1800787 was modulated by TS in relation to CLT. Negative associations between TS and CLT were seen in the major C allele carriers.

**Table 4:** *FGB*, gene polymorphisms' interactions with biomarkers of iron status in relation to clot properties.

**Interaction**	**Interaction *p*-value**	**Genotype**	***r***	***p*-value**	***r***	***p*-value**
**Serum iron in relation to slope**					
***FGB*****:** rs4463047	0.001 (0.003)	TT TC CC	−0.08 −0.07 0.12	0.01 0.23 ^*^	−0.06 0.08 0.40	0.06 0.21 ^*^
**Ferritin in relation to lag time**					
***FGB*****:** rs2227388	0.0009 (0.001)	GG AG AA	−0.03 −0.30 −0.07	0.50 >0.05 0.78	0.03 −0.30 −0.20	0.50 >0.05 0.50
**TS in relation to slope**					
***FGB*****:** rs4463047	0.0008 (0.0004)	TT TC CC	−0.08 0.08 0.47	0.01 0.21 ^*^	−0.06 0.09 0.72	0.06 0.16 ^*^
**TS in relation to CLT**					
***FGB*****:** rs1800787	0.001 (0.002)	CC CT TT	0.02 −0.08 −0.25	0.60 0.55 ^*^	0.02 −0.06 −0.40	0.63 0.63 ^*^

*First, r and p-value are for a GLM interaction adjusted for age, LDL-C, HbA1c, GGT, and BMI; and the second with additional adjustment for fibrinogen. Key: A, adenine; C, cytosine; FGB, fibrinogen beta chain; G, guanine; rs, reference sequence; T, thymine; TS, transferrin saturation*.

* *Stratification resulted in too few individuals to perform analysis*.

## Discussion

To date, limited information is available on the associations between iron and fibrinogen concentration, clot structure and properties (46). Furthermore, reports available on severe iron status abnormalities and their association with fibrinogen and blood clot data are scarce. However, based on the limited available literature, it seemed plausible that fibrinogen and clot properties are affected negatively in both iron deficiency and overload. Therefore, to complement the linear relationship detected by correlation analysis, we created tertiles of the iron status markers in addition to clinical cutoffs used for ferritin and compared the subgroups. In this study, we confirmed that inverse linear relationships exist between iron biomarkers and fibrinogen as well as clot characteristics and detected new linear relationships that have not been reported previously. Only fibrinogen concentrations tended to be higher in the iron-adequate and overload groups than in the deficient group (based on the clinical cut-offs for ferritin values). We did not observe any other U- or J-shaped relationships. This work also contributes to the field in a novel way by considering genetics.

Total fibrinogen but not γ' fibrinogen negatively correlated with serum iron concentrations, with total and γ' fibrinogen decreasing over the serum iron tertiles. The negative relationship between serum iron and total fibrinogen observed here is in agreement with the findings of others on Black South Africans (47, 48). Here, despite not detecting a correlation between ferritin and fibrinogen, fibrinogen tended to be higher (borderline significant) in the iron-adequate and overload groups than in the deficient group. Studies are conflicting regarding the relationship between ferritin and fibrinogen, with one finding no association (49) and others observing a positive association in healthy men and patients with stable angina, myocardial ischemia and unstable angina (50, 51). Reasons for this inconsistent relationship observed between ferritin and fibrinogen are unclear, but could be due to the different target populations that were investigated (apparently healthy vs. having CVD). A weak negative correlation between fibrinogen and TS was detected after *post hoc* correction for covariates. The inverse association was further supported with fibrinogen decreasing over the TS tertiles and being lower in the TS category corresponding to adequate iron status. The negative relationship of TS and total fibrinogen we report contrasts with data from a study involving patients with chronic kidney disease (52). Here we noted novel negative relationships between γ' fibrinogen and serum iron/transferrin. %γ' fibrinogen negatively correlated with transferrin, albeit weakly, and decreased over the transferrin tertiles.

Regarding the clot properties, lag time demonstrated a weak positive association with transferrin (after adjustment for fibrinogen). Additionally, lag time differed between TS iron status categories (before adjustment for fibrinogen), being lower in the iron-deficient group than those with adequate status. CLT correlated negatively with ferritin and was longer in the first than in the third ferritin tertile and shorter in the iron overload group than those with an adequate status based ferritin. CLT was shorter in those with adequate status based on TS cut-offs. In kidney disease patients, D-dimer, a fibrin degradation product indicating coagulation activation and fibrinolysis, seems to be augmented in those with anemia (53). In 2017, a case study reported elevated D-dimer in a patient presenting with anemia after iron sucrose infusion (54). Experimental studies have shown iron-mediated increased coagulation (8, 22, 24, 55). Hydroxyl free radicals are produced by poorly chelated iron ions accumulating in the circulation and converting soluble human fibrinogen into an insoluble and plasmin-resistant polymer. Fibrin clots of this polymer are resistant to enzymatic degradation (56). The proposed biological mechanism for iron-mediated hypercoagulability is not just via oxidative stress, but also involves direct binding of iron to fibrinogen, causing conformational changes in this glycoprotein (57). Lipinski et al. added iron ions (ferrous chloride) to whole blood and observed enhanced fibrin fiber formation with thrombin, delayed fibrinolysis in a concentration-dependent manner and more densely matted fibrin deposits (23). The ferrous ions caused the appearance of dense deposits of matted fibrin, similar to those observed in the plasma of stroke patients (55). In iron overload, a decrease in the time of onset of coagulation was observed (22). These *in vitro* findings do not seem to apply to the epidemiological setting. Here, fibrinogen concentrations seemed to be higher and CLT shorter in those with iron-overload (as defined by ferritin and TS concentrations) than those with adequate status or in the first ferritin tertile than the third, whereas no other marker of clot properties differed after Bonferroni correction. Because we observed a negative association between serum iron and total and γ' fibrinogen and no relationship between ferritin and fibrinogen and the CLT relationship remained after adjusting for fibrinogen, we attribute the increased lysis rate to other factors that also influence fibrinolysis.

To our knowledge, we are the first to relate a unique set of variables reflecting fibrinogen functionality—that is, total fibrinogen or γ' fibrinogen concentrations as well as the kinetics of clot formation, structural properties and fibrinolysis—with iron status biomarkers and to take the genetic make-up of individuals into account. Here we report that certain *FGB* genetic variants modified the association of iron biomarkers with fibrin clot characteristics. Iron biomarkers modulated the influence of the SNPs so that, for the majority of the study population, having adequate iron status was beneficial in respect of clot structure/properties. Optimal iron was evidently even more beneficial for a minority group harboring specific variant alleles. The interactions observed require further replication and functional validation for a mechanistic understanding. However, we know that iron status has the ability to perturb normal patterns of DNA methylation (58) and influence epigenetic regulatory mechanisms (59). We speculate that these methylation changes can translate into altering fibrinogen expression due to conformational changes at the *FGB*: rs4463047 (downstream), *FGB*: rs2227388 (5'upstream) and *FGB*: rs1800787 (5' upstream) loci, leading to the interaction we observed. However, after additional adjustment for fibrinogen the interactions remained. Therefore, future research should investigate the mechanisms behind the interactions observed per locus; here we simply demonstrate the nuanced relationships of iron biomarkers and clot structure/properties due to genetic factors and that they are probably not due to changes in fibrinogen concentration.

Overall, we observed increasing iron biomarkers to be beneficial in terms of fibrinogen and clot properties and did not detect iron-hypercoagulability as previous researchers did (8). However, we acknowledge certain limitations. It is possible that we did not have enough participants presenting with extreme iron deficiency or overload, because we included ostensibly healthy individuals who did not have pronounced iron status abnormalities. To interpret iron status, we accounted for the degree of inflammation by conducting a sensitivity analysis and by adjusting for inflammation; however, maintaining statistical power became problematic when dealing with the study population in terms of particular genotypes. Whereas, two *FXIII* SNPs were included in this study, we could not quantify FXIII levels to provide additional evidence for the association of FXIII levels with fibrin clot structure. Serum transferrin receptor (TfR) concentration and the ratio of serum TfR to serum ferritin may be another indicator of iron deficiency to consider in future (5). Even though hemoglobin is a late indicator of iron deficiency, because it is widely used in clinical settings, its inclusion may aid healthcare workers in giving medical advice. Additionally, viscoelastic testing complimentary to the already collected clot properties could be investigated in future research. We believe that, even with these limitations and the observational nature of the study design, these results still contribute usefully to current knowledge on this topic. Moreover, we have highlighted the idiosyncrasies that result from the genetic differences in how iron status modulates fibrinogen concentration and functionality by investigating blood clot structure and properties.

## Conclusion

We show relationships between fibrinogen, fibrin formation and fibrinogen lysis and biomarkers of iron status that are modulated by certain fibrinogen SNPs. CVD and iron status have both been associated with increased mortality and morbidity. Fibrinogen and fibrinogen functionality may form part of the mechanisms linking these conditions. Therefore, in terms of overall health both iron insufficiency and overload should be detected and treated. Whether manipulation of dietary iron status to influence fibrinogen and fibrin blood clot formation in the presence of certain genetic characteristics can reduce CVD, still needs to be determined. Healthcare workers could potentially use these findings, when enough evidence such as we provide here has accumulated, to modify iron status of individuals to ultimately reduce CVD in the emerging field of personalized medicine.

## Data Availability Statement

The data analyzed in this study is subject to the following licenses/restrictions: The data that support the findings of this study are available upon reasonable request and with the permission of the Health Research Ethics Committee of North-West University and the principal investigator of the PURE-SA-NW study, at North-West University's Africa Unit for Transdisciplinary Health Research. Requests to access these datasets should be directed to lanthe.kruger@nwu.ac.za.

## Ethics Statement

The studies involving human participants were reviewed and approved by North-West University Health Research Ethics Committee (NWU-00016-10-A1 and NWU-00034-17-A1-02). The patients/participants provided their written informed consent to participate in this study.

## Author Contributions

PR: statistical analysis and interpretation of data, drafting and finalizing the manuscript, and final approval. CN-R: study concept, acquisition of data and analysis, drafting, finalizing and critical revision of the manuscript, final approval, and study supervision. ZL-L: data generation and interpretation, critical revision of the manuscript, and final approval. MP: obtained funding, acquisition of data, interpretation of data, critical revision of the manuscript, final approval, and study supervision. All authors contributed to the article and approved the submitted version.

## Conflict of Interest

The authors declare that the research was conducted in the absence of any commercial or financial relationships that could be construed as a potential conflict of interest.

## Publisher's Note

All claims expressed in this article are solely those of the authors and do not necessarily represent those of their affiliated organizations, or those of the publisher, the editors and the reviewers. Any product that may be evaluated in this article, or claim that may be made by its manufacturer, is not guaranteed or endorsed by the publisher.
